# Recommendations for the management of hyperkalemia in patients receiving renin–angiotensin–aldosterone system inhibitors

**DOI:** 10.1007/s11739-023-03427-0

**Published:** 2023-09-29

**Authors:** Luca De Nicola, Pietro Manuel Ferraro, Andrea Montagnani, Roberto Pontremoli, Francesco Dentali, Giorgio Sesti

**Affiliations:** 1 Nephrology Unit, Advanced Medical and Surgical Sciences Department, University of Campania “Luigi Vanvitelli”, Naples, Italy; 2 U.O.S. Terapia Conservativa della Malattia Renale Cronica, Dipartimento di Scienze Mediche e Chirurgiche, Fondazione Policlinico Universitario A. Gemelli IRCCS, Rome, Italy; 3 Dipartimento Universitario di Medicina e Chirurgia Traslazionale, Università Cattolica del Sacro Cuore, Rome, Italy; 4 Department of Internal Medicine, Hospital Misericordia, Grosseto, Italy; 5 Department of Internal Medicine, University of Genoa, Genoa, Italy; 6 Department of Medicine and Surgery, Insubria University, Varese, Italy; 7 Department of Clinical and Molecular Medicine, University of Rome-Sapienza, Rome, Italy; 8 Section of Nephrology, Department of Medicine, Università degli Studi di Verona, Verona, Italy; 9 IRCCS Ospedale Policlinico San Martino, Genova, Italy

**Keywords:** Chronic kidney disease, Cardiovascular disease, Hyperkalemia, Patient-centered care, Potassium binders, Renin–angiotensin–aldosterone inhibitors

## Abstract

Hyperkalemia is common in clinical practice and can be caused by medications used to treat cardiovascular diseases, particularly renin–angiotensin–aldosterone system inhibitors (RAASis). This narrative review discusses the epidemiology, etiology, and consequences of hyperkalemia, and recommends strategies for the prevention and management of hyperkalemia, mainly focusing on guideline recommendations, while recognizing the gaps or differences between the guidelines. Available evidence emphasizes the importance of healthcare professionals (HCPs) taking a proactive approach to hyperkalemia management by prioritizing patient identification and acknowledging that hyperkalemia is often a long-term condition requiring ongoing treatment. Given the risk of hyperkalemia during RAASi treatment, it is advisable to monitor serum potassium levels prior to initiating these treatments, and then regularly throughout treatment. If RAASi therapy is indicated in patients with cardiorenal disease, HCPs should first treat chronic hyperkalemia before reducing the dose or discontinuing RAASis, as reduction or interruption of RAASi treatment can increase the risk of adverse cardiovascular and renal outcomes or death. Moreover, management of hyperkalemia should involve the use of newer potassium binders, such as sodium zirconium cyclosilicate or patiromer, as these agents can effectively enable optimal RAASi treatment. Finally, patients should receive education regarding hyperkalemia, the risks of discontinuing their current treatments, and need to avoid excessive dietary potassium intake.

## Introduction

Hyperkalemia is common in clinical practice, especially in patients with cardiovascular diseases (e.g., hypertension, heart failure [HF] or coronary artery disease), renal impairment, and/or diabetes [1] and can have fatal consequences by causing cardiac arrhythmias [2].

Hyperkalemia can be caused by medications used to treat cardiovascular disease, particularly renin–angiotensin–aldosterone system inhibitors (RAASis) [1]. Since these agents are recommended to reduce target organ damage and improve major clinical outcomes in patients with hypertension, HF, diabetes, and chronic kidney disease (CKD) [3–9], it is important to determine how to prevent and manage hyperkalemia in a way that does not negatively impact treatment for the patient’s underlying condition.

The aim of the current narrative review is to describe the epidemiology, etiology, and consequences of hyperkalemia and to recommend strategies for the prevention and management of hyperkalemia, with a focus on guideline recommendations while recognizing the gaps or differences between guidelines.

## Epidemiology of hyperkalemia

There is no internationally agreed definition of hyperkalemia, and the thresholds for defining the severity of hyperkalemia differ between guidelines (Table 1) [1, 10–13]. The European Society of Cardiology (ESC) defines hyperkalemia as serum potassium levels > 5.0 mmol/L [1], which is the most common definition; this level, while not in general a risk factor for cardiac arrhythmias, should trigger a more careful and frequent monitoring because it poses, per se, a higher risk of severe hyperkalemia [1, 10–13]. Depending on the definition, the estimated prevalence of hyperkalemia is 2–3% in the general population and 1–10% in hospitalized patients [14]. Hyperkalemia prevalence increases with age [15, 16], worsening renal function [15–18], the presence of diabetes or HF [15, 16], a history of myocardial infarction [15], the use of RAASis [15, 17], or resistant hypertension treated with mineralocorticoid receptor antagonists (MRAs) [19–21]. Therefore, the prevalence of hyperkalemia can be as high as 40–50% in these high-risk groups [18, 22]. Conversely, use of sodium-glucose cotransporter 2 inhibitors (SGLT2is) decreases the risk of hyperkalemia in RAASi-treated patients with CKD, likely because of the kaliuresis induced by osmotic diuresis, the increased distal sodium delivery and the preservation of renal function [21, 23].

**Table 1:** Definitions of hyperkalemia in different sets of guidelines

Guideline	Serum potassium levels that define hyperkalemia, mmol/L		
	Mild	Moderate	Severe
American Heart Association, 2005 [11]	5.1–5.9	6.0–7.0	> 7.0
US National Kidney Foundation, 2014 [13]	5.1–5.5	5.6–6.0	> 6.0
Canadian Cardiovascular Society, 2016 [12]	5.0–5.5	5.6–5.9	> 5.9
European Society of Cardiology, 2018 [1]	5.1–5.4	5.5–6.0	> 6.0
UK Renal Association, 2020 [10]	5.5–5.9	6.0–6.4	≥ 6.5

## Clinical impact of hyperkalemia

Cohort studies from Denmark and Italy have indicated that patients with HF and CKD tend to have multiple recurrent episodes of hyperkalemia, with the period between hyperkalemia episodes decreasing with each subsequent event [24–26]. A database analysis of US patients with CKD who were not on dialysis reported that patients spent between 13.2 and 32.4% of their time in a hyperkalemic state during a median of 2.76 years of follow-up [27]. Indeed, the more severe the renal impairment, the longer patients spent in a hyperkalemic state [27].

Serum potassium levels have a U-shaped relationship with all-cause mortality in both healthy and high-risk individuals [27–32], with a significant increase in mortality for patients with serum potassium levels < 4.0 mmol/L and ≥ 5.0 mmol/L [28]. While diabetes, HF, and CKD increase the risk of mortality associated with hyperkalemia, the risk is highest when all three are present [28].

Hyperkalemia also increases the risk of hospitalization among patients with HF or CKD [24, 25], as well as major adverse cardiovascular events (MACE) in patients with CKD [30]. These events contribute to significant economic costs and poor clinical outcomes. In Italy, it has been estimated that maintaining normal serum potassium levels (i.e., normokalemia) in a patient with CKD would save more than €16,000 over the patient’s lifetime, mainly due to a delayed need for dialysis (by an average of 2.29 years) and a longer life expectancy (by an average of 1.79 years) [33]. Indeed, by maintaining normokalemia, clinicians do not need to down-titrate or discontinue RAASis, which translates into a delayed need for dialysis and longer life expectancy.

Among patients undergoing dialysis, the presence of hyperkalemia significantly increases the risk of hospitalization [34, 35], emergency department visits [34], cardiovascular mortality [35, 36], all-cause mortality [34–36], and sudden death [37, 38]. The risk of sudden death is also 2.7-fold higher in patients with a predialytic serum potassium ≥ 6.0 mmol/L than in those with serum potassium < 6.0 mmol/L [38]. The risk of sudden death or hospitalization associated with hyperkalemia is increased at the end of a long interdialytic interval (i.e., 60–72 h, such as over weekends), consistent with the adverse outcomes that result from serum potassium accumulation [34, 37].

## Clinical impact of renin–angiotensin–aldosterone system inhibitor withdrawal

For patients receiving RAASis, the risk posed by hyperkalemia must be balanced against the risk of avoiding or withdrawing these agents, which have a proven benefit in terms of cardiovascular outcomes. Despite guideline recommendations, a high proportion of patients receiving RAASis in the clinical practice setting receive suboptimal doses [39–42].

Data from the US CHAMP-HF registry show that a minority of patients with HF receive stable target doses of RAASis over 12 months of follow-up; in fact, 73.0% of patients receiving angiotensin-converting enzyme inhibitors (ACEis) or angiotensin II receptor blockers (ARBs) had stable sub-target doses over this time, and 11.5% had a dose reduction or discontinued the ACEi/ARB [40]. For patients on MRAs, the corresponding percentages were 67.3% and 4.4% [40]. Medical reasons were the most common cause of dose decrease or discontinuation [40].

Hyperkalemia is a common reason for reducing the dose or discontinuing RAASis [39, 43]. An analysis of data from a large US database of electronic medical records found that, in patients who developed hyperkalemia during RAASi treatment, the RAASi dose was titrated downwards in 16–21% of events, and RAASi was discontinued in 22–27% of hyperkalemic events [39]. However, both RAASi discontinuation and submaximal doses of RAASis were associated with an increased risk of adverse cardiovascular outcomes (composite) or death over a median of 3.4 years of follow-up [39]. BLITZ-HF, a cross-sectional study in patients with acute or chronic HF, found hyperkalemia to be one of the main factors underlying a lack of RAASi implementation in this population [44]. In a Canadian population-based cohort study, discontinuation of RAASis in patients with hyperkalemia and CKD was associated with an increased risk of dialysis initiation and mortality [45].

In Europe, the ESC-HFA-EORP Heart Failure Long-term Registry showed that hyperkalemia was associated with an increased risk of mortality, as well as an increased risk of discontinuing RAASi treatment [16]. However, hyperkalemia was no longer associated with an increased risk of death after adjustment for RAASi discontinuation, suggesting that treatment withdrawal was the true cause of the increased mortality risk, and hyperkalemia was a marker for treatment discontinuation [16].

Similar findings were reported in a cohort study among outpatients with HF in Italy [46]. In this analysis, hyperkalemia was a common cause of MRA dose reduction or withdrawal in patients with HF. While hyperkalemia per se was not associated with an increased risk of mortality, MRA withdrawal secondary to hyperkalemia was associated with a fivefold increased risk of mortality after adjustment for baseline risk factors [46].

The Randomized Aldactone Evaluation Study (RALES) found that, in patients with HF, the use of spironolactone 25 mg/day was associated with lower mortality rates even for those with moderate hyperkalemia (up to 6.0 mmol/L); at higher potassium levels, the dependent negative effects, including MRA withdrawal, outweighed the cardioprotective efficacy of this drug [47]. These data argue against automatic discontinuation of MRAs when potassium concentrations rise to > 5.0 mmol/L. Nevertheless, patients must be carefully followed for hyperkalemia when treated with MRAs; this concept is supported by a real-world analysis examining trends in the rate of spironolactone prescriptions and the rate of hospitalization for hyperkalemia in ambulatory patients before and after the publication of RALES [48]. The analysis found that following the increase in the prescription rate of spironolactone in HF after publication of RALES, hospitalizations and mortality due to hyperkalemia did actually increase by 4 and 7 times, respectively.

## Guideline recommendations for hyperkalemia

A number of guidelines on the management of cardiorenal diseases contain recommendations on hyperkalemia, including the Kidney Disease: Improving Global Outcomes (KDIGO) guidelines for diabetes management in CKD [9], the KDIGO guidelines for blood pressure management in CKD [8], the ESC HF guidelines [5], and the American College of Cardiology (ACC) HF guidelines [49].

In addition, there are several sets of guidelines specifically on hyperkalemia management, including international consensus recommendations [50], guidelines from the UK Renal Association [10], and the Italian Society of Nephrology (ISN) position paper [14].

## Monitoring serum potassium during renin–angiotensin–aldosterone system inhibitor treatment

The KDIGO guidelines recommend that ACEis or ARBs should be used in patients with advanced CKD, but note that close monitoring of serum potassium is required in patients with CKD to aid early identification of even moderate hyperkalemia, in order to prevent more severe degrees of this complication [8, 9]. The ESC HF guidelines also recommend close monitoring of serum potassium, with optimization of RAASi treatment when potassium levels are 4.5–5.0 mmol/L [5]. Once an elevated serum potassium level has been recorded, it is advisable to repeat the test for confirmation. For example, the UK guidelines recommend repeating the test within 3 days if the initial level was 5.5–5.9 mmol/L and within 1 day if the level was 6.0–6.4 mmol/L [10]. Patients with serum potassium ≥ 6.5 mmol/L should be considered as having acute hyperkalemia and be admitted for immediate assessment and treatment [10].

## Diet

The KDIGO guidelines, the ISN position paper on hyperkalemia, and the Kidney Disease Outcomes Quality Initiative (KDOQI) guidelines on nutrition in patients with CKD all recommend limiting foods rich in potassium among patients with hyperkalemia, but also note the importance of considering the patient’s need for adequate fiber intake [8, 9, 14, 51]. The KDOQI guidelines suggest identifying the most important sources of potassium in the patient’s diet and reducing them, ideally with the assistance of a renal dietician [51]. However, there is generally a lack of high-quality evidence demonstrating the effectiveness of dietary potassium restriction as a management strategy for hyperkalemia [2, 51]. In fact, a low-potassium diet may deprive patients of important nutrients and could conflict with a heart-healthy diet, such as the Dietary Approaches to Stopping Hypertension (DASH) diet [52, 53]. For example, patients on hemodialysis generally have a low intake of fruits and vegetables, but increasing their consumption reduced both the risk of all-cause mortality (by 5.0%) and the risk of death due to non-cardiovascular causes (by 3.0%) [54]. A study by the Centers for Disease Control and Prevention Chronic Kidney Disease Surveillance Team showed that increased consumption of fruits and vegetables led to a slower rate of decline of kidney function among CKD and non-CKD individuals [55]. Patients who are trying to limit their dietary potassium intake may also face many practical barriers and psychosocial issues [56], which means that other potassium-lowering strategies are often needed for effective hyperkalemia management.

## Pharmacological treatment

The ESC HF guidelines recommend initiating pharmacological potassium-lowering treatment with potassium binders as soon as serum potassium exceeds 5.0 mmol/L [5]. RAASi treatment should only be reduced or discontinued in patients whose serum potassium exceeds 6.5 mmol/L. On the other hand, the ACC HF guidelines advocate a higher threshold for identifying hyperkalemia (serum potassium ≥ 5.5 mmol/L) [49]. These guidelines note that potassium binders facilitate the continuation of RAASi treatment, but that the effect of this treatment continuation on cardiovascular outcomes is uncertain [49].

The KDIGO guidelines and international consensus recommendations for hyperkalemia recommend that RAASi treatment should not be de-escalated or discontinued unless alternative measures for hyperkalemia management have been optimized or these measures have failed to normalize serum potassium [8, 9, 50]. Oral supplementation with sodium bicarbonate can be used in patients with metabolic acidosis, and potassium-wasting diuretics may be an option only in patients with extracellular volume expansion [10, 14]. However, prolonged supplementation with sodium bicarbonate may increase the sodium load, which may worsen fluid overload in patients with CKD and HF [10]. Similarly, the use of diuretics requires careful monitoring and management to prevent worsening hypovolemia or renal function with subsequent increase in serum potassium [14].

Most guidelines (i.e., those of the ESC, ACC, international consensus recommendations, and the UK Renal Association) advocate the use of newer potassium binders (i.e., patiromer sorbitex calcium [patiromer] and sodium zirconium cyclosilicate [SZC]) to manage hyperkalemia while maintaining RAASi treatment [5, 10, 49, 50].

## Pharmacological and clinical profile of potassium binders

The earlier generation of potassium binders was potassium exchange resins based on polystyrene, either sodium polystyrene sulfonate (SPS) or calcium polystyrene sulfonate (CPS), which act in the gastrointestinal tract to swap potassium for sodium or calcium, respectively, thereby increasing fecal potassium excretion [57]. While both are effective in reducing serum potassium [58], SPS is more widely used [57]. Both CPS and SPS are associated with gastrointestinal adverse events, particularly constipation, nausea, and vomiting, but also potentially serious adverse events, including ulcers, perforation, and ischemia/thrombosis [58–62]. For example, in a Canadian cohort study among 27,704 elderly patients initiating SPS treatment, serious gastrointestinal adverse events requiring emergency department presentation or hospitalization developed in the first 30 days at a rate of ~ 23 per 1000 patient-years; the risk was highest for gastrointestinal ischemia or thrombosis (fourfold increase in the risk compared with non-use of SPS) [62]. The risk of intestinal necrosis is increased when these agents are used with sorbitol, a common laxative for constipation [2, 57].

As with the older potassium binders, patiromer and SZC are not systemically absorbed but act in the gastrointestinal tract to trap potassium ions for subsequent fecal elimination; however, the newer potassium binding agents show minimal water absorption or swelling in the gastrointestinal tract, resulting in fewer adverse effects [57, 63, 64].

Patiromer consists of a non-absorbed cation exchange polymer with a calcium-sorbitol counter-ion complex that increases stability [63]. While SPS exchanges potassium for sodium ions, patiromer exchanges potassium for calcium ions; therefore, it may be safer than SPS in patients who should avoid even small increases in sodium loads, such as those with severe hypertension, CKD, or HF [63]. Patiromer is designed to be maximally ionized (i.e., have the greatest binding capacity) at the physiological pH of the gastrointestinal tract, where potassium concentrations are the highest [63].

SZC is a microporous zirconium silicate that mimics the action of physiological potassium ion channels [64]. It is highly selective for potassium, with minimal effect on the absorption of other cations such as calcium or magnesium [64]. SZC has a rapid onset of effect (within 1 h) [65], and, therefore, is the preferred option in instances where a relatively rapid reduction in serum potassium levels is needed [66]. By comparison, the onset of action for patiromer is 4–7 h and is not suited to emergency treatment [67].

Both patiromer and SZC have demonstrated their efficacy in short-term correction of serum potassium, as well as maintaining levels within the normokalemic range over the long term, in patients with hyperkalemia (Table 2), including those with CKD, HF, hypertension, or type 2 diabetes [68–80]. However, as noted in the ACC guidelines [49], no data are yet available regarding the impact of patiromer or SZC on cardiovascular outcomes.

**Table 2:** Clinical trials with patiromer sorbitex calcium or sodium zirconium cyclosilicate in patients receiving renin–angiotensin–aldosterone system inhibitors

Study (year)	Design	Patients	*N*^a^	Duration	Key results
*Patiromer*					
PEARL-HF (2010, 2011) [70, 75]	R, DB, PC	CKD and HF receiving background RAASis or BBs, with spironolactone added	105	4 weeks	Significant reduction in K^+^ levels (–0.45 mmol/L; p < 0.001) and significantly lower incidence of K^+^ levels ≥ 5.5 mmol/L (7% vs 24%; p = 0.027) with patiromer vs placebo at Week 4
AMETHYST-DN (2015) [69]	MC, R, OL	T2D and CKD receiving RAASis	306	52 weeks	Significant reduction in K^+^ level among patients with mild or moderate hyperkalemia at Week 4 and maintained for 52 weeks
OPAL-HK (2015) [78]	MC, R, SB, PC	CKD receiving RAASi	243	12 weeks	After 4 weeks of treatment with patiromer, withdrawal led to increase in serum K^+^ level of 0.72 mmol/L (vs 0 mmol/L in patients who stayed on patiromer) at Week 4 and development of serum K^+^ level ≥ 5.5 mmol/L in 60% of patients (vs 15% who stayed on patiromer) by Week 8
AMBER (2019) [68]	MC, R, DB, PC	CKD and persistent HT receiving spironolactone	295	12 weeks	66% in placebo group vs 86% in patiromer group were still on spironolactone at week 12 (*p* < 0.0001), and hyperkalemia (serum K^+^ level ≥ 5.5 mmol/L) was present in significantly more placebo vs patiromer recipients (*p* < 0.001)
DIAMOND (2022) [80]	MC, R, DB, PC	HFrEF and hyperkalemia on RAASi and/or MRA	878	13–43 (median 27) weeks	Significantly lower K^+^ levels (–0.13 mmol/L; p < 0.001), significantly lower incidence of K^+^ levels > 5.5 mmol/L (13.9% vs 19.4%; *p* = 0.006) and significantly fewer MRA dose reductions or discontinuations (13.9% vs 18.9%; *p* = 0.006) with patiromer vs placebo
*SZC*					
HARMONIZE (2014) [73]	MC, R, DB, PC	Outpatients with serum K^+^ level ≥ 5.1 mmol/L (69.8% on RAASi)	258	28 days	SZC achieved normokalemia in 98% of patients at 48 h; SZC 5, 10, or 15 g maintained serum K^+^ level at < 5.1 mmol/L in 80%, 90% and 94% of patients, respectively, versus 46% of placebo recipients at Week 4 (all *p* < 0.001)
HARMONIZE OLE (2019) [76]	OLE	Outpatients with serum K^+^ level 3.5–6.2 mmol/L	123	≤ 337 days	SZC 5 or 10 g maintained serum K^+^ level at ≤ 5.1 and ≤ 5.5 mmol/L in 88.3% and 100% of patients, respectively
ZS-003 (2015) [74]	MC, R, DB, PC	Outpatients with serum K^+^ level 5.0–6.5 mmol/L (66.7% on RAASi)	754	16 days	SZC rapidly and dose-dependently reduced serum K^+^ levels to normokalemic levels within 48 h; normokalemia was maintained in a higher proportion of patients continuing on SZC vs switched to placebo (*p* ≤ 0.008)
DIALIZE (2019) [71]	MC, R, DB, PC	ESRD on dialysis	196	8 weeks	Significantly more SZC than placebo recipients maintained serum K^+^ levels at 4.0–5.0 mmol/L during 3–4 dialysis sessions after long interdialytic interval (41.2% vs 1.0%; *p* < 0.001)
ZS-005 (2019) [77]	MC, OL	Outpatients with serum K^+^ level ≥ 5.1 mmol/L (65% on RAASi)	751	52 weeks	78% of patients receiving SZC achieved serum K^+^ level of 3.5–5.5 mmol/L during the 3-day correction phase, and 99% had a mean serum K^+^ level of 3.5–5.5 mmol/L over Months 3–12
HARMONIZE-GLOBAL (2020) [79]	MC, R, DB, PC	Outpatients with serum K^+^ level ≥ 5.1 mmol/L (76.4% on RAASi)	267	28 days	SZC achieved normokalemia in 89.1% of patients at 48 h; SZC 5 or 10 g maintained serum K^+^ level at 3.5–5.0 mmol/L in 58.6% and 77.3% of patients, respectively, versus 24.0% of placebo recipients on Day 29 (both *p* < 0.001)

*BB* β-blocker, *CKD* chronic kidney disease, *DB* double-blind, *ESRD* end-stage renal disease, *HF* heart failure, *HFrEF* heart failure with reduced ejection fraction, *HT* hypertension, *K*^*+*^ potassium ion, *MC* multicenter, *MRA* mineralocorticoid receptor antagonist, *OL* open-label, *OLE* open-label extension, *PC* placebo-controlled, *R* randomized, *RAASi* renin–angiotensin–aldosterone system inhibitor, *SB* single-blind, *SZC* sodium zirconium cyclosilicate, *T2D* type 2 diabetes

^a^Randomized and/or treated population

Importantly, the newer potassium binding agents allow for the continued use of RAASis at stable or increased doses in most patients [68, 69, 76, 77, 80]. SZC treatment is associated with increases in serum bicarbonate, which can reduce the risk of metabolic acidosis and the need for alkali supplementation (e.g., with sodium bicarbonate) [81]. Patiromer and SZC are both generally well tolerated; common adverse events during patiromer treatment are hypomagnesemia, constipation, diarrhea, abdominal pain, and flatulence [67], while the most common adverse events during SZC treatment are constipation and edema-related events [65]. The latter effect, resulting from the presence of ~ 400 mg of sodium for each 5 g dose of SZC, must be especially considered in patients with non-dialysis CKD and HF [82].

## Recommendations for hyperkalemia management

Clear differences exist between sets of guidelines on hyperkalemia management in patients with cardiorenal disease, and it may be helpful to have a comprehensive and unified guideline that considers the potential for multiple comorbidities in these patients. Based on the available evidence, we advocate that physicians take a proactive approach to hyperkalemia management in clinical practice that focuses on patient identification and recognizes that hyperkalemia is often a long-term condition that needs ongoing treatment.

## Patient identification

Hyperkalemia is simple to diagnose but may go undetected in an outpatient setting because it is usually asymptomatic. Therefore, given the risk of hyperkalemia during RAASi treatment, it is advisable to check serum potassium prior to initiating these treatments, and then regularly throughout treatment [10, 14]. This is particularly important in older patients and those who have a history of hyperkalemia, who are at increased risk of subsequent hyperkalemic events [15, 24–26, 47]. Currently, there are no internationally agreed criteria for the magnitude, duration, and frequency of elevated serum potassium that define chronic hyperkalemia [52].

ESC HF guidelines and the position paper of the Italian ISN recommend starting potassium binders when serum potassium is ≥ 5.0 mmol/L [5, 14], while in the UK, the threshold of serum potassium for the use of potassium binders is higher (> 6.0 mmol/L) [10]. Based on the most recent KDIGO guidelines in diabetic and nondiabetic CKD [9] (personal communication, M Madero), we recommend that potassium binder treatment is initiated with a serum potassium level of at least 5.5 mmol/L confirmed in two tests and after excluding pseudohyperkalemia. Indeed, this is the threshold for initiating potassium binders according to the Italian Drug Agency [83].

## Treatment

All healthcare professionals (HCPs) involved in the management of patients with cardiovascular or renal disease should recognize that hyperkalemia is a predictable and manageable adverse effect of RAASi-containing treatment regimens. Therefore, HCPs need to be familiar with the current guideline recommendations for the management of hyperkalemia, as well as of the patient’s cardiorenal disease.

Based on the known benefits of RAASis in patients with cardiorenal disease, clinical practice guidelines such as those from the ESC consistently recommend treating chronic hyperkalemia first before reducing the dose or discontinuing RAASi treatment if serum potassium levels exceed 6.5 mmol/L [5, 10, 14, 50]. The only exception may be a temporary interruption of RAASis during an acute intercurrent illness, such as sepsis, hypovolemia, or acute kidney injury [10]. Alternatively, the ACC guidelines recommend discontinuing MRAs if serum potassium levels cannot be maintained < 5.5 mmol/L [49]. Nevertheless, a recent randomized open-label crossover trial in patients with CKD suggested that adding an SGLT2i to MRA therapy provides increased kidney and cardiovascular protection and reduces the risk of MRA-related hyperkalemia [84].

**Fig. 1:**
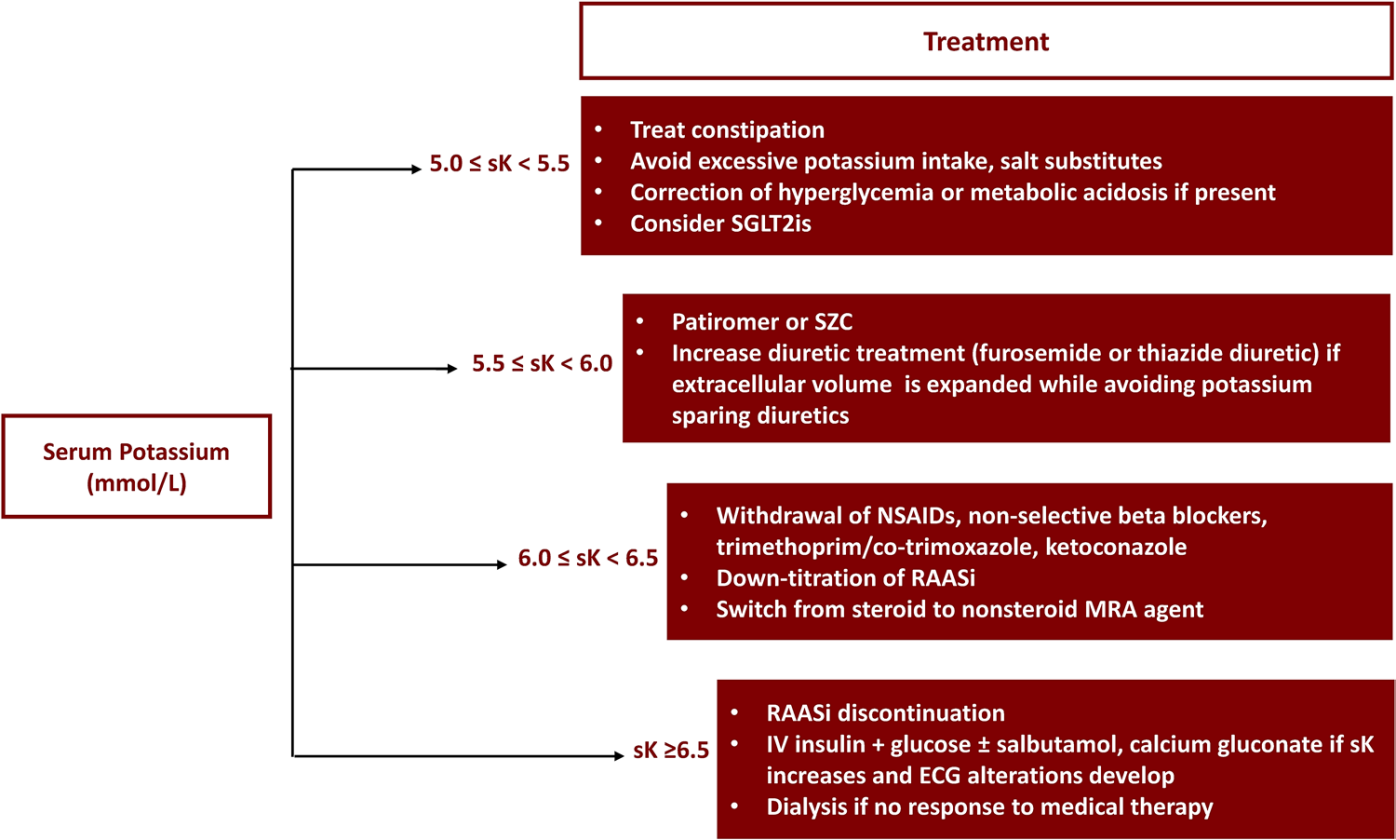
Pragmatic step-by-step intervention for the treatment of hyperkalemia in patients with hyperkalemia. *ECG* electrocardiogram, *IV* intravenous, *MRA* mineralocorticoid receptor antagonist, *NSAID* non-steroidal anti-inflammatory drug, *RAASi* renin–angiotensin–aldosterone system inhibitor, *sK* serum potassium, *SGLT2i* sodium-glucose cotransporter 2 inhibitor, *SZC* sodium zirconium cyclosilicate

Treatment should involve the use of newer potassium binders (i.e., SZC or patiromer), based on evidence that these agents can allow patients to effectively achieve normokalemia while optimizing RAASi treatment [68, 69, 76, 77]. An optimal serum potassium level of 4.0–4.5 mmol/L has been suggested by the ISN position paper because these levels are associated with the lowest mortality rate [14]; however, we suggest a target serum potassium of 4.0–4.9 mmol/L, which may be more feasible in clinical practice. There is no consensus (considering clinical trial evidence) on the specific treatment of hyperkalemia tailored to a given potassium level; therefore, in Fig. 1, we propose a pragmatic step-by-step approach to hyperkalemia in non-dialysis CKD patients that summarizes indications by current guidelines and position papers [2, 8, 10, 14, 49, 52] (personal communication, M Madero), where each additional step contains the interventions indicated in the previous steps. This approach can be implemented in all outpatients with CKD and chronic hyperkalemia receiving RAASi therapy, regardless of the main comorbidities (hypertension, diabetes, HF). In-hospital treatment of acute severe hyperkalemia, i.e., in the setting of emergency units, cardiac arrest, and resuscitation, goes beyond the scope of this review; this topic has been extensively addressed elsewhere [10]. It is worth noting that SZC and patiromer might also allow the effective treatment of hyperkalemia in some patients who are not deemed to be candidates for dialysis treatment [85].

Patients should receive education on hyperkalemia, as well as the risks of discontinuing their current RAASi treatments, and the need to avoid excessive dietary potassium intake. In patients with CKD who experience hyperkalemia frequently, the recommended potassium intake is < 3 g/day—corresponding to an approximate urinary potassium excretion of < 78 mmol/24 h [86]—while maintaining a healthy diet rich in fruits and vegetables [86, 87]. If patients are suitable for potassium binder treatment, they must understand how important it is to follow this therapy, as a successful combination of optimal RAASi plus potassium binder can avoid/delay the need for dialysis and other serious clinical outcomes. Patients and clinicians need to understand that liberalization/normalization of their diet is possible, but that potassium excess should be still avoided.

## A plea for stakeholder engagement and research

Institutions and payers need to be aware of the risk of hyperkalemia and RAASi down-titration, and they should promote, through all available channels, the effective management of hyperkalemia without RAASi dose adjustment wherever possible. HCPs need to encourage all colleagues to learn the guideline recommendations and apply them in clinical practice. Closer cross-specialist collaboration will help to optimize outcomes for people with cardiorenal disease. Patient groups and associations need to educate patients of all risks associated with hyperkalemia and RAASi down-titration, and should encourage patient and caregiver education. Scientific societies need to be a reliable partner of the above-mentioned stakeholders and encourage a patient-centric approach to hyperkalemia management. Finally, further research is needed, including head-to-head comparisons of individual potassium binders and real-world clinical studies of patients who are treated according to the evidence-based recommendations, such as the TRACK study (ClinicalTrials.gov identifier: NCT05408039), which aims to increase understanding of hyperkalemia management, treatment models and decision making for the management of hyperkalemia.

## Data Availability

Data sharing not applicable to this article as no datasets were generated or analyzed during the current study.
